# Elastic Properties of Reinforced Body-Centered Cubic Lattice Structures

**DOI:** 10.3390/ma19132852

**Published:** 2026-07-03

**Authors:** Mauro Giacalone, Sara Mantovani

**Affiliations:** “Enzo Ferrari” Engineering Department, University of Modena and Reggio Emilia, 41125 Modena, Italy; sara.mantovani@unimore.it

**Keywords:** lattice structures, Additive Manufacturing, Finite Element methods, elasticity

## Abstract

Lattice structures have gained particular interest in the last years, because of the spread of Additive Manufacturing, which allowed their production with ease. These structures may be used as functionally graded materials for lightweighting in structural components, or they can be tailored to match the mechanical properties of bone tissue for orthopedic implants. To reduce the computational time and costs of structural simulation and optimization, this study presents a numerical homogenization to determine the main elastic constants of the BCCz lattice, over its relative density. Numerical simulations are carried out on a lattice with a nominal geometry, made from a homogeneous isotropic material. Results present charts and interpolating functions of the elastic constants of the lattice, over its relative density, that may help the designer in tailoring the lattice structure to the desired applications. Results show that the BCCz presents a substantial influence of the load direction on the mechanical properties, with the z direction showing superior properties than the transverse direction. This makes the BCCz lattice ideal for those structures where the main load directions are easily predictable.

## 1. Introduction

Cellular lattice structures have spread widely in recent years, along with the use of Additive Manufacturing (AM). These structures have proven their handiness, which has been proven in various fields of application. Research has shown efficient applications of lattice structures for heat exchange between fluids [1,2], for thermal dissipation [3] or insulation [4,5], or acoustic insulation as well [6,7].

Besides these applications, cellular structures have also been used for lightweighting in structural components.

The literature presents examples of automotive components integrating lattice structures [8,9,10]. Abdi et al. [8] optimize a brake pedal for maximum stiffness and weight reduction. They introduce a lattice structure to improve the resistance of the foot pad. Yin et al. [9] designed a lattice structure to be inserted as a core for composite sandwich panels and then used these panels to substitute an automotive hood. Their numerical simulations evidenced up to a 25% weight reduction from the original hood, while guaranteeing reasonable safety from a head impact on the hood. Mantovani et al. [10] show the optimization of an automotive steering support. The use of AM and a lattice structure led to the reduction of almost half of the mass of the original casted support.

The design of orthopedic implants benefits from the use of lattice structures [11]. That is because the open pores within the structure help bone tissue to penetrate them [12] and because the elastic modulus of the reticular structures may be adapted to that of bone tissue to reduce bone tissue recession. Deng et al. [13] tested, in vivo, the growth of bone tissue within scaffolds made of various lattice structures, underlining those unit cells which led to better integration.

Structural components with lattice structures may be designed with the help of Topology Optimization techniques [14]. Panesar et al. [15] showed that a lattice structure with a graded density coming from Topology Optimization outperforms a uniform lattice. These Topology Optimization methods adopt the homogenization method; the constitutive equations of the lattice structure are expressed as functions of the density of the lattice, as presented by Cheng et al. [16].

The homogenization method consists of determining the physical and elastic properties of a homogeneous material, which is structurally equivalent to the lattice. This strategy is not only applied to Optimization, but it may also help in reducing the computational costs of design and Finite Element simulation of structural components with lattice structures. In this sense, the literature shows a wide range of methods.

The stiffness and strength of reticular lattices may be obtained by modeling each element of the lattice structure with classical beam theory. Gibson and Ashby [17,18] distinguish between stretch-dominated and bending-dominated lattices. Lake and Klang [19] determine the beam section sizes to obtain globally isotropic lattice structures. Martinsson and Babuška [20] present a technique that uses the Fourier expansion on the static and thermal equilibrium equations of a given truss structure. Then, the equations of the homogenized equivalent continuous material are obtained by letting the size of the lattice tend to zero. De Felice and Sorrentino [21] use this framework to derive the dynamic equilibrium of in-plane lattices.

The use of beam theory leads to good results with few manageable calculations, but lacks some precision when the relative density of the lattice increases, and this is due to some reasons. First, in these models, shear deformation is oftentimes neglected, and this cannot be neglected when the thickness of the beams becomes considerable. Then, the joints among beams are considered rigid, while in actual lattices, they are slightly compliant. Finally, the geometry of the beams are approximated with perfect cylinders, which eventually overlap at the joints between the beams. This results in some error when calculating the lattice relative density, and this error increases with the relative density itself.

In any case, these models yield some perfectly fine results when the relative density remains under 10 percent. When dealing with higher densities, the Finite Element (FE) method may be implemented to determine the mechanical properties of a lattice. Sun and Vaidya [22] consider a Representative Volume Element (RVE), performing linear static FE simulations on such element with various loading conditions and derive the elastic constants from the elastic energy resulting from the FE simulations. The use of adequate boundary conditions is crucial in this sense, to make sure that the RVE in the FE model is surrounded by an infinite domain, full of the same RVEs. Mizzi et al. [23] present a boundary condition scheme to guarantee the correct loading and constrain of the RVE.

A similar work was presented by Andreassen and Andreasen [24] who worked on a 2D multi-material composite. They provide a Matlab 2019B routine that obtains the equivalent thermal, structural, electrical, and fluid-flow properties of the composite.

Numerical homogenization methods were first applied to fiber-reinforced composites, but are also perfectly applicable to lattices, which are composite materials themselves. The only requirement is the correct identification of the RVE, which is the lattice unit cell.

This contribution applies a numerical homogenization technique like that of Sun and Vaidya [22] to determine the elastic properties of a body-centered cubic lattice, reinforced with beams along one edge of the cube, which is typically called BCCz. The homogenization technique will make use of general-purpose periodic boundary conditions similar to those presented by Mizzi et al. [23] and is meant to cover all the densities between 0 (i.e., absence of lattice material) and 1 (i.e., lattice volume completely filled with constituent material).

The results will report some laws connecting the relative density to each elastic constant, like what was presented by Cheng et al. [16]. These laws may hopefully help designers in simplifying the design and optimization of structural components, which may adopt this lattice structure.

## 2. Materials and Methods

### 2.1. Lattice Unit Cell and Density

**Figure 1:**
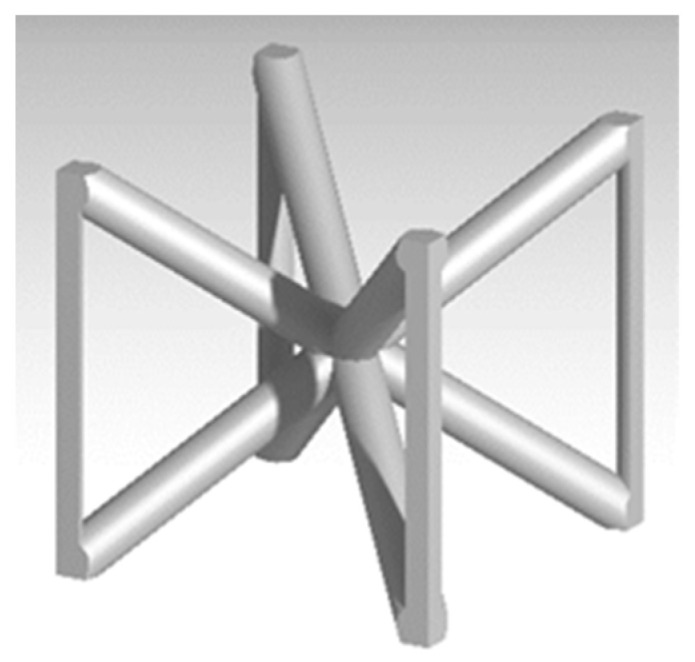
BCCz unit cell under exam.

The lattice under exam is the Body-Centred Cubic, reinforced along Z direction (BCCz), and is shown in Figure 1. The behavior of this unit cell varies according to the load direction. A tensile load along the Z direction puts the reinforcement beams under tension, and the unit cell seems to act as a stretch-dominated structure, as per the–Gibson-Ashby classification [17]. Conversely, under a shear load or a transverse axial load, the beams crossing at the center of the cube bend, leading to the typical bending dominated behavior. This generally diminishes the lattice strength and stiffness.

Therefore, the geometry of this unit makes it suitable for those applications where a high stiffness-to-weight ratio is needed in one clear direction only, like what happens in honeycomb structures. Designers could still use this lattice in components with multiaxial loads but need to address the design with particular care, as the stiffness and strength is not uniform along the three directions.

A CAD model of the unit cell was generated with an edge size (*l*) of 1 mm and a diameter (*d*), which varied between 0.05 mm and 0.70 mm. Catia v5 was used to generate 14 unit cells and to calculate the volume occupied by the beams in the unit. This volume returned automatically the relative density of the lattice (ρ/ρ_s_). Results are summarized in Table 1.

**Figure 2:**
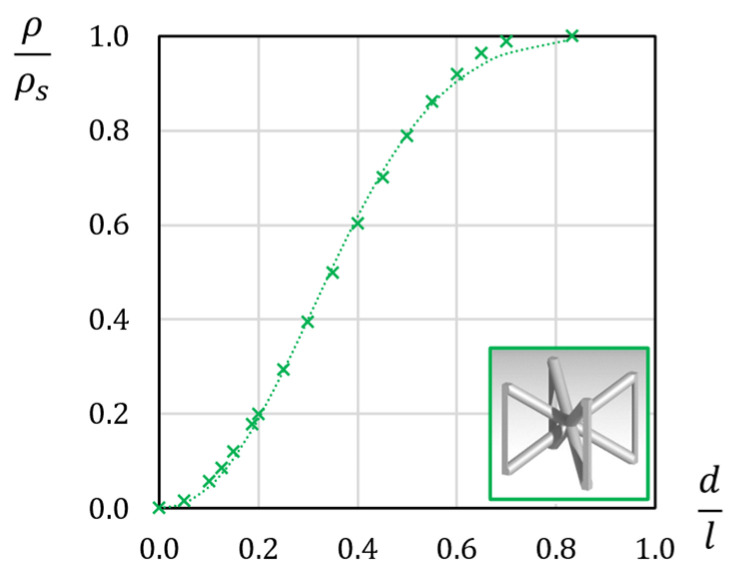
ρ/ρ_s_ over *d*/*l* for the BCCz unit cell. The dotted line represents the approximating law (1).

Figure 2 shows the relation between ρ/ρ_s_ and *d*/*l*. Numerical results are approximated with an S-shaped curve. As predicted by the Gibson–Ashby model [17], at the lowest diameters, ρ/ρ_s_ increases quadratically with *d*/*l*, because the volume of the beams has a quadratic increase with beam diameters.

At higher diameters, the overlap of the beams at the joints increases, slowing the increase of ρ/ρ_s_. At *d*/*l* between 0.3 and 0.5 ρ/ρ_s_ is linear with the diameter. Then, the curve flattens when ρ/ρ_s_ approaches 1. Calculations from the CAD model of the unit cell show that ρ/ρ_s_ becomes one when *d*/*l* is equal to √(2/3) (about 0.816).

The approximating curve in Figure 2 is obtained with the following formula:

(1) $$\frac{\rho }{\rho_{s}}=1-exp\left[-4.63{\left(\frac{d/l}{\sqrt{2/3}}\right)}^{2.2}\right],$$

which approximates the measurements with an R^2^ of 0.9988. As shown in Figure 2, the interpolation law (1) yields reasonable approximation of ρ/ρ_s_ at *d*/*l* below 0.6. The lowest accuracy is encountered at *d*/*l* between 0.75 and 0.8, where the ρ/ρ_s_ of the lattice is between 0.9 and 1.

### 2.2. Numerical Homogenization

The homogenization technique aims at determining a continuous, fictional material which is structurally equivalent to the BCCz lattice. For such material, the constitutive equations apply:

(2) σ=Cε,

*C* is the compliance matrix, which has, in general, 21 independent terms:

(3) $$C=\left[\begin{array}{cccccc} c_{11} & c_{12} & c_{13} & c_{14} & c_{15} & c_{16}\\ c_{21} & c_{22} & c_{23} & c_{24} & c_{25} & c_{26}\\ c_{31} & c_{32} & c_{33} & c_{34} & c_{35} & c_{36}\\ c_{41} & c_{42} & c_{43} & c_{44} & c_{45} & c_{46}\\ c_{51} & c_{52} & c_{53} & c_{54} & c_{55} & c_{56}\\ c_{61} & c_{62} & c_{63} & c_{64} & c_{65} & c_{66} \end{array}\right],$$

however, most materials in use in engineering have way less independent elastic constants. To give an example, orthotropic materials like composites reinforced with fibers may be modeled with 9 elastic constants, while isotropic materials need two independent constants only.

It is assumed in this study that the constituent material of the BCCz lattice is isotropic, which applies to metal alloys or polymers. Lake and Klang [19] showed that rotational symmetries within the lattice geometry have a great influence on the homogenization. In their study, they explain how any geometrical symmetry will also apply to the constitutive law of the homogeneous equivalent material. They also provide some useful results according to some common symmetries.

**Figure 3:**
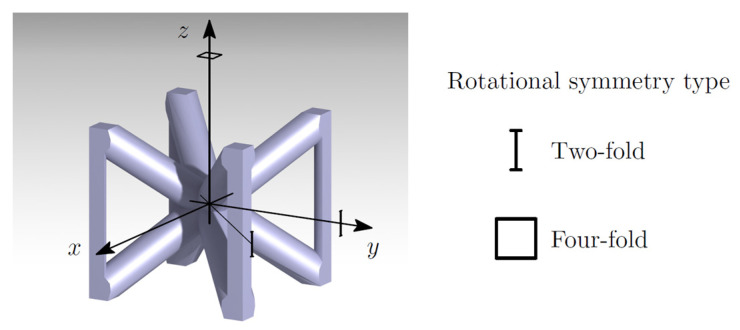
ρ/ρ_s_ over *d*/*l* for the BCCz unit cell.

The BCCz lattice has three main rotational symmetries, as shown in Figure 3, which shows the rotational symmetries of the BCCz lattice. A 90-degree symmetry is found along the *z* axis, while on the *xy* plane, we find four axes of 180-degree symmetry. With this symmetry scheme, the homogeneous equivalent material is orthotropic with respect to x, y, and z. Furthermore, the constitutive matrix has only 6 independent elastic constants [19], as shown in (4):

(4) $$C=\left[\begin{array}{cccccc} c_{11} & c_{12} & c_{13} & 0 & 0 & 0\\ c_{12} & c_{11} & c_{13} & 0 & 0 & 0\\ c_{13} & c_{13} & c_{33} & 0 & 0 & 0\\ 0 & 0 & 0 & c_{44} & 0 & 0\\ 0 & 0 & 0 & 0 & c_{44} & 0\\ 0 & 0 & 0 & 0 & 0 & c_{66} \end{array}\right].$$

these constants are obtained combining two Young moduli (*E_x_*, *E_z_*), two Shear moduli (*G_zx_*, *G_xy_*), and two Poisson’s ratios (*ν_zx_*, *ν_xy_*):

(5) $$\begin{array}{c} c_{11}=\frac{E_{x}\left(1+\nu_{xz}\nu_{zx}\right)}{1-{\nu_{xy}}^{2}-\left(2\nu_{xy}+2\right)\nu_{xz}\nu_{zx}},\\ c_{12}=\frac{E_{x}\left(\nu_{xy}+\nu_{xz}\nu_{zx}\right)}{1-{\nu_{xy}}^{2}-\left(2\nu_{xy}+2\right)\nu_{xz}\nu_{zx}},\\ c_{33}=\frac{E_{z}\left(1-\nu_{xy}\right)}{1-\nu_{xy}-2\nu_{xz}\nu_{zx}},\\ c_{13}=\frac{E_{z}\nu_{xz}}{1-\nu_{xy}-2\nu_{xz}\nu_{zx}},\\ b_{44}=G_{zx},\\ b_{66}=G_{xy}. \end{array}$$

To simplify the notation, a third Poisson’s ratio (*ν_xz_*) was adopted in Equation (5). However, *ν_xz_* id obtained from the other independent constants:

(6) $$\frac{\nu_{xz}}{E_{x}}=\frac{\nu_{zx}}{E_{z}}.$$

Finite Element (FE) simulations were adopted to calculate the constants within *C*. These simulations imposed a unit strain to the lattice and keeping all the other strain components to zero. For example, when applying a unit strain *ε_x_*, the equations become the following:

(7) $$\left\{\begin{array}{c} \sigma_{x}\\ \sigma_{y}\\ \sigma_{z}\\ \tau_{yz}\\ \tau_{zx}\\ \tau_{xy} \end{array}\right\}=C\left\{\begin{array}{c} 1\\ 0\\ 0\\ 0\\ 0\\ 0 \end{array}\right\},$$

therefore, the stress components that keep this deformed state are equal to the first column of *C*:

(8) $$\left\{\begin{array}{c} \sigma_{x}\\ \sigma_{y}\\ \sigma_{z}\\ \tau_{yz}\\ \tau_{zx}\\ \tau_{xy} \end{array}\right\}=\left\{\begin{array}{c} c_{11}\\ c_{21}\\ c_{31}\\ c_{41}\\ c_{51}\\ c_{61} \end{array}\right\}.$$

By repeating the process for all six strain components, one obtains all the coefficients of *C*.

With the help of elastic symmetries, the full C may be obtained with only four FE simulations, for example *ε_x_*, *ε_z_*, *γ_zx_*, and *γ_xy_*. The remaining columns of *C* are just a rearrangements of columns obtained with these four simulations, as in (4).

### 2.3. FE Simulation Setup

**Figure 4:**
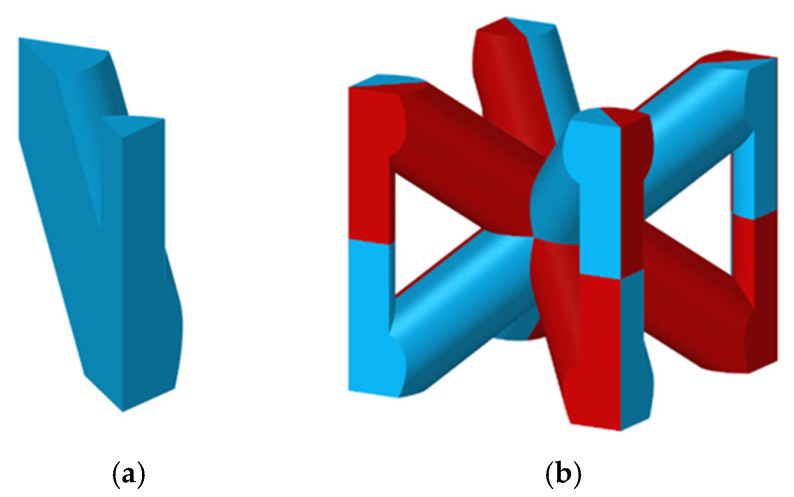
(**a**) Fundamental patch of the unit cell; (**b**) FE model of the complete BCCz unit cell (the colors distinguish all the patches within the cell).

The FE modeling of the lattice was created considering one single unit cell and the symmetries within the cell itself. At first, a patch, constituting 1/16 of the unit cell was extracted from the CAD model of each unit cell. This patch was then discretized with first order, tetrahedral elements (Figure 4a). Then, the patch underwent reflections and rotations to obtain the complete unit cells (Figure 4b). After a mesh convergence test, the average mesh size for these models was chosen to be about 0.01 mm for all the FE models of the unit cells. Altair^®^ Hypermesh was adopted for meshing and the reflections. The numerical campaign was made of 14 FE models, all at different ρ/ρ_s_ spanning between 0 and 1.

The FE simulations in this numerical campaign were performed on only one unit cell, imagining that such a unit cell is immersed in an infinite lattice domain. This condition is guaranteed by imposing periodic boundary conditions at the external faces of the cell [22,23].

**Figure 5:**
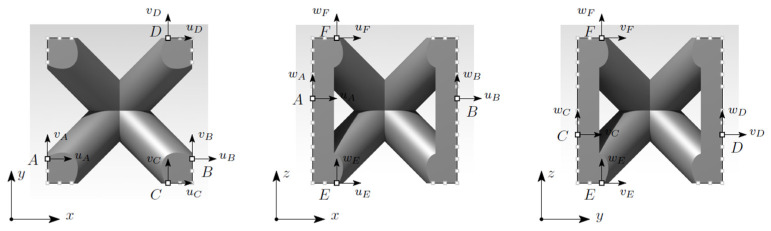
Schematic representation of boundary nodes A–F, and their displacements within the three orthogonal planes.

Looking at Figure 5, which evidences three generic boundary nodes A, D, and F on the positive faces of the unit cell, paired with the two corresponding nodes on the negative faces, B, C, and E, the deformation of the unit cell may be linked to the displacements of such nodes.

Under the assumption that a single unit cell is much smaller than the lattice domain containing the cell, the strain components of the lattice may be approximated with the following finite differences:

(9) $$\begin{array}{c} \varepsilon_{x}=\frac{\Delta u}{\Delta x}=\frac{u_{B}-u_{A}}{l}.\\ \varepsilon_{y}=\frac{\Delta v}{\Delta y}=\frac{v_{D}-v_{C}}{l},\\ \varepsilon_{z}=\frac{\Delta w}{\Delta z}=\frac{w_{F}-w_{E}}{l},\\ \gamma_{yz}=\frac{\Delta v}{\Delta z}+\frac{\Delta w}{\Delta y}=\frac{v_{F}-v_{E}}{l}+\frac{w_{D}-w_{C}}{l},\\ \gamma_{zx}=\frac{\Delta w}{\Delta x}+\frac{\Delta u}{\Delta z}=\frac{w_{B}-w_{A}}{l}+\frac{u_{F}-u_{E}}{l},\\ \gamma_{xy}=\frac{\Delta u}{\Delta y}+\frac{\Delta v}{\Delta x}=\frac{u_{D}-u_{C}}{l}+\frac{v_{B}-v_{A}}{l}, \end{array}$$

by adding three arbitrary conditions on the rigid rotations of the lattice,

(10) $$\begin{array}{c} \frac{\Delta u}{\Delta y}=\frac{\Delta v}{\Delta x},\\ \frac{\Delta v}{\Delta z}=\frac{\Delta w}{\Delta y},\\ \frac{\Delta w}{\Delta x}=\frac{\Delta u}{\Delta z}, \end{array}$$

the displacements at the boundary nodes of the cell become the following:

(11) $$\begin{array}{c} u_{B}=u_{A}+l\varepsilon_{x},\\ v_{B}=v_{A}+\frac{1}{2}l\gamma_{xy},\\ w_{B}=w_{A}+\frac{1}{2}l\gamma_{zx},\\ u_{D}=u_{C}+\frac{1}{2}l\gamma_{xy},\\ v_{D}=v_{C}+l\varepsilon_{y},\\ w_{D}=w_{C}+\frac{1}{2}l\gamma_{yz},\\ u_{F}=u_{E}+\frac{1}{2}l\gamma_{zx},\\ v_{F}=v_{E}+\frac{1}{2}l\gamma_{yz},\\ w_{F}=w_{E}+l\varepsilon_{z}. \end{array}$$

These conditions coincide with those of periodic displacements. One may verify by imposing an arbitrary strain *ε_y_* while imposing all the remaining strain components to be zero.

The kinematic conditions (11) were imposed by using multi-DOF constraints on the FE model of the unit cell. Nodes on the positive faces (i.e., nodes B, D, and F) are the dependent nodes of these multi-DOFS constraints, while their counterparts on the negative faces (i.e., nodes A, C, and D) remain independent. Then, three auxiliary nodes are introduced outside of the unit cell as further independent nodes in the multi-DOF constraints. The se nodes are introduced to impose arbitrary strains on the unit cell, and this is done by imposing an adequate displacement to such auxiliary nodes.

**Figure 6:**
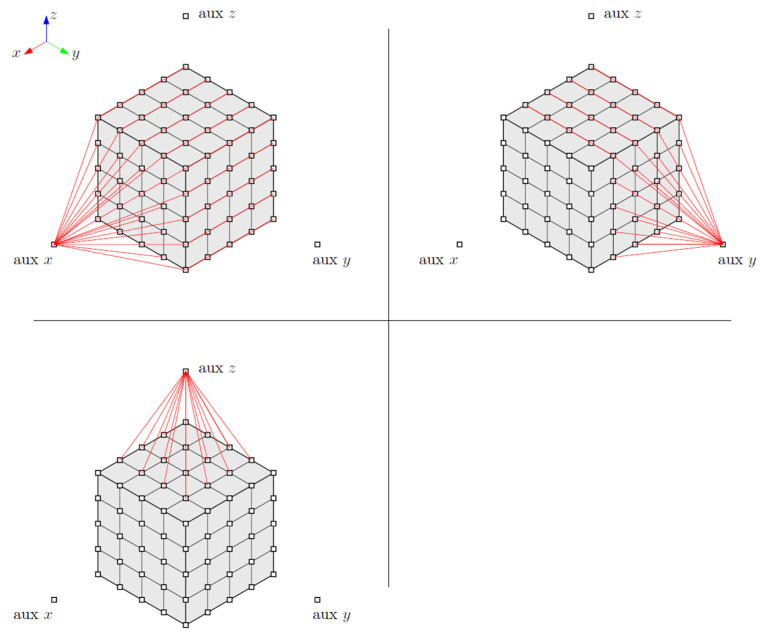
Example of the multi-DOF constraints involving the faces of the unit cell normal to the three coordinate axes.

Each multi-DOF constraint involves a single pair of nodes and the respective auxiliary nodes. A single constraint is applied to each pair of nodes on the outer faces and for each translational degree of freedom of the nodes. Figure 6 shows a schematic representation of the proposed boundary conditions to a cubic FE model. Some nodes are excluded in order not to constrain some degrees of freedom twice.

Displacements are imposed on the auxiliary nodes to enforce the desired strains to the unit cell. For example, a unit strain *ε_x_* was applied by imposing a displacement along *x* equal to *l* to aux x node, while fixing all the other displacements at the aux nodes to zero.

This scheme has two major advantages: first, it may be easily applied to generic hexahedral unit cells. Then, once all the displacements at the auxiliary nodes are imposed, the unit cell is constrained with respect to the three rigid rotations. The three rigid translations may be constrained by fixing one node within the unit cell.

The reaction forces at the auxiliary nodes, divided by the area of the face of the unit cell, returned the average stress on the lattice unit cell. These results were extracted to determine the equivalent elastic constants of the unit cell, according to Equation (5).

All the models in this numerical campaign adopted a homogeneous, isotropic constituent material, with a Young Modulus (*E_s_*) equal to 1 and Poisson’s ratio (*ν_s_*) equal to 0.3.

MSC^®^ Marc 2020.1 was used to run the FE simulations on this campaign. Four simulations were performed on per each ρ/ρ_s_, according to the mentioned elastic symmetry. The complete stiffness matrix *C* was composed by rearranging the results. The process was repeated at all ρ/ρ_s_, with the addition of two more results:

1. ρ/ρ_s_ = 0: In this case, the lattice degenerates in an empty volume, with no structural material. In this case, *E*/*E_s_* and *G*/*E_s_* along all the directions and planes are assumed to be zero, while *ν* is undetermined;
2. ρ/ρ_s_ = 1: Here, the lattice coincides with the constituent material, as well as the equivalent elastic constants. Therefore *E*/*E_s_* = 1, *G*/*E_s_* = 1/2(1 + *ν_s_*) and *ν* = *ν_s_*.

## 3. Results and Discussion

**Figure 7:**
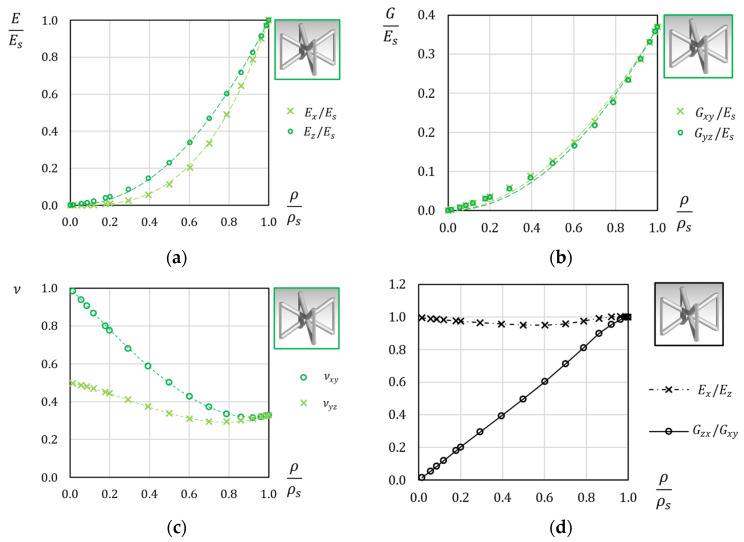
(**a**) Relative Young moduli over the density; (**b**) relative shear moduli over the density; (**c**) Poisson’s ratios of the unit cells (dashed lines represent approximating functions in Equation (11)); (**d**) comparison between elastic moduli and shear moduli.

Figure 7 shows the results of the numerical campaign, arranged as the equivalent elastic constants over ρ/ρ_s_. Contrary to the predictions of the Gibson–Ashby model [17], the Young modulus *E_z_*/*E_s_* does not show a linear increase, even if the lattice is a stretch-dominated structure when loaded along *z*. In any case, the reinforcing beams add a significant contribution to the relative modulus along *z* (*E_z_*/*E_s_*), which remains sensibly higher than the modulus in the transverse direction (*E_x_*/*E_s_*). This is underlined in Figure 7d, which shows the ratio between the Young Moduli of the lattice. From the numerical results, it appears that $E_{x}/E_{z}$ is nearly linear with ρ/ρ_s_, meaning that the lower the ρ/ρ_s_, the higher the difference between the two Young moduli. The relative Young moduli then converge when ρ/ρ_s_ increases above 0.9. This is because at such high densities, the lattice resembles a solid material with some small, residual holes, and no beam-like feature may be distinguished.

The difference in stiffness evidenced by Figure 7a,d suggests the use of this lattice with a finely-tuned orientation, within a component where the main loading directions are well known, or at least easily calculated.

Figure 7b shows also very little difference between the two shear moduli *G_xy_*/*E_s_* and *G_xz_*/*E_s_*, meaning that the reinforcement beams have little influence on these constants. The ratio between $G_{zx}$ and $G_{xy}$ is shown in Figure 7d across all ρ/ρ_s_. Numerical results show that the two shear moduli remain quite close together, with a minimum ratio of 0.949 at a ρ/ρ_s_ of 0.6. This means that the two shear moduli differ from one another by less than 5.1% across all the observed densities.

Therefore, the shear of this lattice unit is mainly due to the diagonal beams crossing at the center of the cube. It is also worth noticing that at a low ρ/ρ_s_, the shear moduli are higher than the elastic modulus along *x* or *y*. This is due to the fact that when a shear is imposed on the lattice, the diagonal beams are loaded mainly under axial and shear loads, while an axial stress along x or y, puts these same beams under shear and bending.

**Figure 8:**
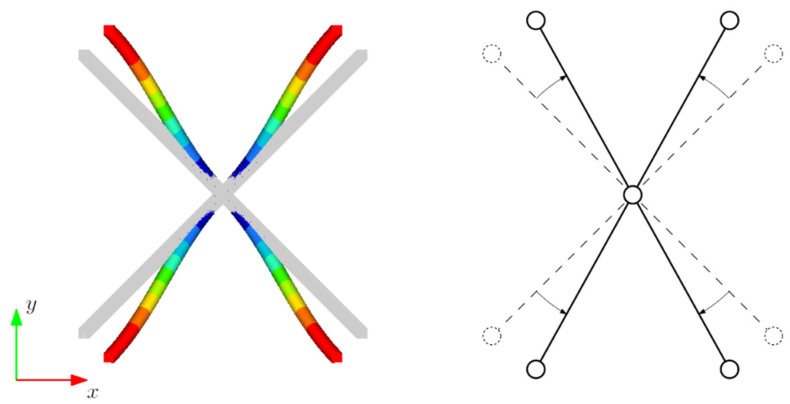
In-plane deformation of a BCCz cell (ρ/ρ_s_ = 0.015) under a uniform stress along *x*.

Figure 7c shows the two homogenized Poisson’s ratios of the lattice. The two *ν* eventually converge to *ν_s_* when ρ/ρ_s_ equals 1, but show a much different behavior when ρ/ρ_s_ is lower. The in-plane Poisson’s ratio *ν_xy_* tends to 1 as ρ/ρ_s_ tends to zero. This is due to the in-plane deformation of the unit cell when loaded axially along *x* or *y*, as shown in Figure 8. As said before, the cell is purely bending dominated in these cases, and when ρ/ρ_s_ is very low, the bending stiffness of the beams becomes much lower than their axial stiffness. When the unit cell is loaded along x or y, the diagonal beams with little to no axial stretch. If we view this deformation from the top, we notice that the diagonal beams have overall slightly rotated around the center of the unit cell, like what would happen if the beams were pinned with one another.

When ρ/ρ_s_ increases, the contribution of shear and axial deformation on the beams comes into play, reducing secondary in-plane deformations.

The Poisson’s ratio *ν_yz_* involves the reinforcing beams, which kind of attenuates transverse deformations, even at a very low ρ/ρ_s_. In fact, *ν_yz_* tends to 0.5 when ρ/ρ_s_ tends to 0. The increase in ρ/ρ_s_ leads to a decrease in *ν_yz_*, with a minimum of about 0.3 at ρ/ρ_s_ equal to 0.7.

The numerical results may be approximated with a continuous function, which may be used in gradient-based optimization [16]. A power law was chosen for relative Young moduli and shear moduli. This law is extremely common for Solid, Isotropic Material with Penalization and may be used to extend the method to orthotropic materials. On the other hand, third-order polynomials are well suited to fit the evolution of the Poisson’s ratios:

(12) $$\begin{array}{c} \frac{E_{x}}{E_{s}}={\left(\frac{\rho }{\rho_{s}}\right)}^{3},\\ \frac{E_{z}}{E_{s}}={\left(\frac{\rho }{\rho_{s}}\right)}^{2.12},\\ \frac{G_{yz}}{E_{s}}=\frac{1}{2\left(1+\nu_{s}\right)}{\left(\frac{\rho }{\rho_{s}}\right)}^{2.03},\\ \frac{G_{xy}}{E_{s}}=\frac{1}{2\left(1+\nu_{s}\right)}{\left(\frac{\rho }{\rho_{s}}\right)}^{1.91},\\ \nu_{yz}=0.508{\left(\frac{\rho }{\rho_{s}}\right)}^{3}-0.454{\left(\frac{\rho }{\rho_{s}}\right)}^{2}-0.223\left(\frac{\rho }{\rho_{s}}\right)+0.5,\\ \nu_{xy}=0.432{\left(\frac{\rho }{\rho_{s}}\right)}^{3}+0.015{\left(\frac{\rho }{\rho_{s}}\right)}^{2}-1.120\left(\frac{\rho }{\rho_{s}}\right)+1. \end{array}$$

The coefficients of the approximating functions in Equation (12) were obtained with the least squared methods. Equation (12) reports an R^2^ of 0.9995 for $E_{z}/E_{s}$, 0.9997 for $E_{x}/E_{s}$, 0.9964 for $G_{yz}/E_{s}$, and 0.9964 for $G_{xy}/E_{s}$.

These interpolating functions may help the designer to reduce the computational cost for the simulation of structural components, which include this lattice structure.

It is important to note that this work performed numerical simulations on a lattice with a nominal geometry. An actual lattice may be manufactured with some geometrical and dimensional inaccuracies, according to how close these structures are to the technological limit of the adopted machines. The simplest way to account for such inaccuracies is to test these structures experimentally, finding a factor to penalize (or to amplify) the elastic moduli found above.

In addition, the numerical campaign performed here considered a uniform diameter for all the beams within the lattice. Some further studies may be carried out to find the elastic constants of the lattice with different diameters for the beams or for a group of beams.

This same framework may also be applied to determine the static or fatigue resistance of the lattice under three-dimensional loads. The unit cell may be loaded with uniform stress, and the maximum equivalent stress may be calculated within the beams in the lattice. Then, these equivalent stresses may be linked to the average stress on the lattice to determine a yield criterion. This criterion may then be confirmed with some extensive experimental studies.

## 4. Conclusions

This paper presented a numerical homogenization for a body-centered cubic lattice reinforced with beams along one direction (BCCz). Finite Element models of the unit cell across all relative densities were obtained with three-dimensional elements.

Periodic boundary conditions were introduced to simulate the unit cell as insert in an infinite lattice domain. In this sense, a general-purpose periodic boundary conditions scheme was presented.

The Young moduli, the shear moduli, and the Poisson’s ratios of the BCCz lattice were obtained across all relative densities and were approximated with analytical functions of the relative densities with a correlation coefficient (R^2^) of 0.9964 or above. Designers may make use of these results to test various densities in the design of their structural components including this cellular structure. The homogenized equivalent material may be adopted to simplify the design and simulation of structural components adopting such a structure. In addition, the proposed equations may be adopted for a density-based Topology optimization.

Results underline the major mechanical properties of this lattice along the direction of the reinforcing beams (i.e., *z* in this case). This suggests that the proposed lattice offers its best performance when aligned with the main loading directions.

## Figures and Tables

**Table 1:** Relative density results at various beam diameters.

*d*/*l*	ρ/ρ_s_
0.05	0.015
0.10	0.056
0.15	0.119
0.20	0.200
0.25	0.293
0.30	0.394
0.35	0.499
0.40	0.603
0.45	0.701
0.50	0.788
0.55	0.861
0.60	0.920
0.65	0.963
0.70	0.988

## Data Availability

The original contributions presented in this study are included in the article. Further inquiries can be directed to the corresponding author.
